# Antibacterial and Bonding Properties of Universal Adhesive Dental Polymers Doped with Pyrogallol

**DOI:** 10.3390/polym13101538

**Published:** 2021-05-11

**Authors:** Naji Kharouf, Ammar Eid, Louis Hardan, Rim Bourgi, Youri Arntz, Hamdi Jmal, Federico Foschi, Salvatore Sauro, Vincent Ball, Youssef Haikel, Davide Mancino

**Affiliations:** 1 Department of Endodontics, Faculty of Dental Medicine, Strasbourg University, 67000 Strasbourg, France; youri.arntz@unistra.fr (Y.A.); vball@unistra.fr (V.B.); youssef.haikel@unistra.fr (Y.H.); davidemancino@icloud.com (D.M.); 2 Department of Biomaterials and Bioengineering, INSERM UMR_S 1121, Strasbourg University, 67000 Strasbourg, France; 3 Department of Endodontics, Faculty of Dental Medicine, Damascus University, Damascus 0100, Syria; ammarendo89@gmail.com; 4 Department of Restorative Dentistry, Saint-Joseph University, Beirut 11072180, Lebanon; louis.hardan@usj.edu.lb (L.H.); rim.bourgi@hotmail.com (R.B.); 5 ICube Laboratory, UMR 7357 CNRS, Mechanics Department, University of Strasbourg, 67000 Strasbourg, France; jmal@unistra.fr; 6 Department of Endodontics, Faculty of Dentistry, Oral & Craniofacial Sciences, Floor 22 Tower Wing, Guy’s Dental Hospital, London SE1 9RT, UK; federico.foschi@kcl.ac.uk; 7 Peninsula School of Medicine and Dentistry, Plymouth University, Plymouth PL4 8AA, UK; 8 Department of Therapeutic Dentistry, I.M. Sechenov First Moscow State Medical University, 119146 Moscow, Russia; salvatore.sauro@uchceu.es; 9 Dental Biomaterials and Minimally Invasive Dentistry, Department of Dentistry, Cardenal Herrera-CEU University, C/Santiago Ramón y Cajal s/n, Alfara del Patriarca, 46115 Valencia, Spain

**Keywords:** universal adhesive, antibacterial activity, bond strength, pyrogallol, polymer–dentin interface

## Abstract

This study investigated the antibacterial activity, bond strength to dentin (SBS), and ultra-morphology of the polymer–dentin interface of experimental adhesive systems doped with pyrogallol (PY), which is a ubiquitous phenolic moiety that is present in flavonoids and polyphenols. A universal adhesive containing 4-META and 10-MDP was used in this study. PY behaves as an antioxidant and anti-cancerogenic agent and it was incorporated into the adhesive at different concentrations (0.5 and 1 wt.%). The antibacterial activity and SBS were analyzed and the results were statistically analyzed. The ultra-morphology of the polymer–dentin interface was assessed using scanning electron microscopy (SEM). At 24 h, a lower antibacterial activity was observed for the control adhesive compared to those with 0.5% and 1% PY. No difference was seen in SBS between the three groups at 24 h. After 6 months, the SBS of the 0.5% PY adhesive was significantly lower than the other tested adhesives. The specimens created with 1% PY adhesive presented a higher bond strength at six months compared with that found at 24 h. No morphological differences were found at the polymer–dentin interfaces of the tested adhesives. Pyrogallol may be incorporated into modern universal adhesive systems to preserve the polymer–dentin bonding interface and confer a certain degree of antibacterial activity.

## 1. Introduction

Despite novel technologies and constant developments in polymer chemistry, there is still a lack of information regarding the bonding performance of modern universal adhesive systems [1]. Currently, these types of adhesive systems represent an advanced choice for dental practitioners, as they can be used in self-etch, etch-and-rinse, or a selective enamel-etching mode [2,3]. However, the bonding efficiency of universal adhesives is still questioned, as the durability and stability of the dentin–adhesive interface are limited, especially when used in etched dentin [4,5,6].

Indeed, adequate bond strength to dentin is generally immediately achieved, whereas issues of longevity related to nanoleakage at the polymer–dentin interface still characterize modern adhesive systems [7]. Such a compromised long-term performance is the result of a complex adhesive composition that is characterized by hydrophilic and hydrophobic components that are all placed in a simplified single-bottle system [8,9].

It is important to consider that during carious removal and just before bonding procedures, bacteria can remain within the dentin substrate, smear layer, in dentinal tubules, and at the dentin–enamel junction [10]; accordingly, recurrent caries and subsequent hypersensitivity could occur [11]. Therefore, the use of antibacterial agents has been proposed after cavity preparation to inhibit bacterial growth [12]. Indeed, the incorporation of the antibacterial agents, such as quaternary ammonium methacrylate [12], methacryloyloxydodecylpyridinium bromide (MDPB) [13], dimethylaminododecyl methacrylate (DMADDM) [14], chlorhexidine [15], or silver nanoparticles in dental adhesives [16] could provide a new perspective to combat such a recurring clinical challenge [17].

Fundamentally, the bonding with the dentin substrate relies on the creation of an interdiffusion zone, also called the “hybrid layer,” which is responsible for the micromechanical adhesion of a composite restoration [18]. Thus, the “hybrid layer” is a mixture of collagen, hydroxyapatite, resin monomers, and residual solvents, and its ultimate stability depends on the resistance of the individual components to degradation *phenomena* [19]. In general, the greater the overall quality of the hybrid layer, the better the longevity of the polymer–dentin bond strength [20]. Since higher mechanical properties and lower bacterial colonization are desired, the use of antibacterial agents in adhesive procedures has gained interest [21,22]. They are successful in protecting the tooth–adhesive interface from microleakage, while providing eradication of residual bacteria in the oral cavity [23].

**Figure 1:**
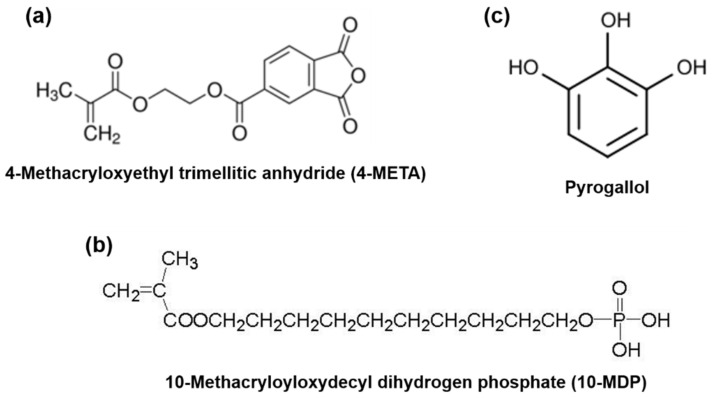
Chemical structure of the acidic monomers used in Iperbond Max and pyrogallol: (**a**) 4-META, (**b**) 10-MDP, and (**c**) pyrogallol.

Iperbond Max (Table 1) is a single-bottle universal adhesive system that contains 4-META and 10-MDP acidic monomers (Figure 1), which act as powerful structural agents and guarantee a tenacious bond to both dentin and enamel [24].

Polyphenols play an interesting role in the oral cavity against many diseases, and they are used in many dental applications [21,25]. Pyrogallol (Figure 1c), previously known as “vegetable tannin,” is a ubiquitous phenolic moiety that is present in flavonoids and polyphenols in a variety of edible plants, such as cacao, nuts, vegetables, and fruit peels [26]. This molecule behaves as an antioxidant and anti-cancerogenic agent. Chemically, pyrogallol consists of benzene-1,2,3-triols and due to the localized hydroxyl groups, its capability to rapidly and robustly form hydrogen bonds and hydrophobic interactions is clearly evident. Moreover, pyrogallol possesses a strong affinity to a variety of proteins [27]. The oxidation of two hydroxyl groups adjacent to the reactive “quinone” form under physiological and weak basic conditions, leading to antioxidant effects, is a further feature of pyrogallol. The resulting quinone can then react with amine and thiol groups, allowing for the covalent modification of biomolecules via Michael addition or a Schiff base formation [28]. Considering the effort devoted to evaluating the chemical structures of numerous pyrogallol-containing molecules, to our knowledge, there is no study available on pyrogallol-modified universal adhesives.

The purpose of this in vitro study was to investigate the antibacterial activity, bond strength to dentin, and ultra/morphology of the polymer-dentin interface of experimental universal resin adhesive systems doped with pyrogallol (PY). The first null hypothesis was that the addition of pyrogallol would not increase the antibacterial activity of the universal adhesive and the second one was that the pyrogallol would have no significant impact on the bond strength of the universal adhesive to dentin.

## 2. Materials and Methods

### 2.1. Materials

A resin adhesive system, Iperbond Max (Itena Clinical, Paris, France) was modified by adding different weight percentages (0.5% and 1% *w*/*w* of PY (ref. P0381-250G, purity higher than 98% from HPLC data), Sigma Aldrich, Saint-Quentin-Fallavier, France) (Table 1). The unmodified adhesive was used as the control group. In addition, 1 mL of the adhesive was placed in a 2 mL Eppendorf tube (Trefflab, Degersheim, Switzerland) using a micropipette. PY powder was placed using a spatula and was weighted using a high-precision balance (Ohaus, Pioneer Analytical, Nänikon, Switzerland). Subsequently, the PY powder (0.5% and 1%) was added to the adhesive in the Eppendorf tube and mixed using a high shear mixer (Fisher Scientific, Illkirch, France) in a dark room at 2000 rpm for 10 min [29] in order to get a well-mixed PY–adhesive solution. The homogenization of the PY resin and complete dissolution were confirmed and it was used only if no crystals were observed under an optical stereomicroscope [30,31].

All the tested adhesives were stored in the dark at 4 °C and once applied onto the dentin, they were light-cured for 20 s using an LED curing system (Luxite Lampe LED, ITENA Clinical, Paris, France).

### 2.2. Antibacterial Activity

#### 2.2.1. Bacterial Strain

*Streptococcus mutans* (*S. mutans*, CIP103220) was cultured using Brain Heart Infusion (BHI) (Panreac Applichem ITW Reagents, Hessen, Germany). The inoculum used for this experiment was prepared using an overnight culture of *S. mutans* in BHI broth at 37 °C. The obtained bacterial suspension was measured to an optical density (OD) of 0.5 at 600 nm with a spectrophotometer (Biorad, Schiltigheim, France) for further usage.

#### 2.2.2. Agar Diffusion Test (ADT)

The agar diffusion tests were performed as described in a previous study [32]. Three agar-filled Petri dishes containing 25 mL of BHI agar were used. They were used to evaluate the antibacterial activity of the different weight percentages (0%, 0.5%, and 1%) of PY incorporated in the resin adhesives. A total of 100 µL of the bacterial medium were spread homogeneously onto the Petri dishes. Four wells in each petri dish (3.0 mm in diameter and 3.0 mm in depth) were performed by removing the agar using a punch (3 mm, PFM medical, Köln, Germany). The first three wells were filled with the adhesive + (0, 0.5, or 1%) PY, respectively, while the fourth well was left unfilled, which represented a control group. All the agar Petri dishes were incubated at 37 °C for 24 h. The inhibition zones after 24 h of incubation were measured in millimeters using a digital caliper (Dexter, Elkhart, IN, USA); they were determined as half the diameter of the inhibition zone minus the well’s diameter [32].

#### 2.2.3. Direct Contact Test (DCT)

Immediately after mixing, 21 µL of each different adhesive (*n* = 3) was placed in a 2 mL Eppendorf tube (Trefflab, Degersheim, Switzerland). One milliliter of the bacterial medium was also injected into each Eppendorf tube. In the positive control group, the bacterial medium was injected into the Eppendorf tube with the presence of no adhesive. Subsequently, the Eppendorf tubes were incubated for 24 h at 37 °C under constant stirring at 600 rpm.

After each incubation period, ten microliters from each tube were assessed using 10-fold serial dilutions up to 10^3^ in BHI medium and then 100 μL of the solution was spread homogeneously onto a plate. These latter plates were incubated at 37 °C for 24 h. After incubation, the colonies on the plate were counted and their colony-forming units/milliliter (CFU/mL) were determined.

### 2.3. Release Kinetics of PY and the pH Measurements

**Figure 2:**
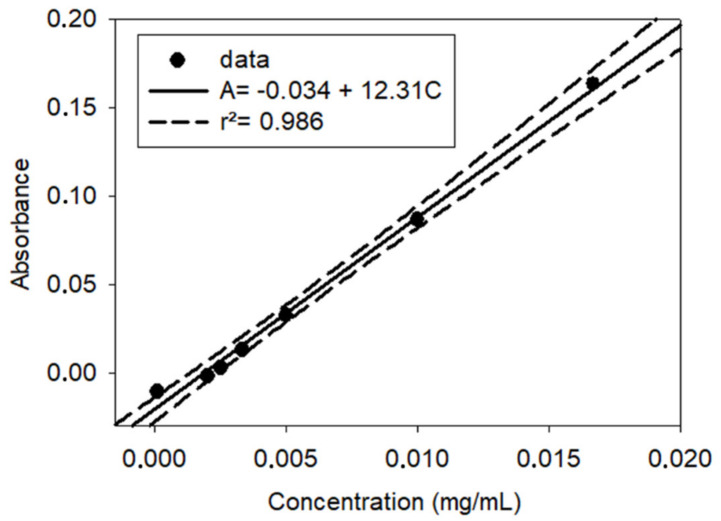
The calibration curve for pyrogallol at 267 nm (r^2^ = 0.986).

The calibration curve for PY was performed (Figure 2). A stock solution of PY (0.1 mg·mL^−1^) was prepared in distilled water. This solution was gradually diluted between 2 and 100 times with distilled water. Its absorption spectra were then measured using a double-beam mc^2^ spectrophotometer (SAFAS, Monaco, Monaco) to establish a calibration curve that allowed for the quantification of PY release from the different adhesives. The measurement cuvette (Quartz, 1 cm path length) was filled with the PY solution, whereas the reference cuvette was filled with distilled water.

Two specimens of each adhesive (0%, 0.5%, and 1%) containing 150 mg of each composite were immersed in 10 mL of distilled water using a glass bottle. After 1, 3, 6, and 24 h, 0.5 mL of each supernatant solution above the sealers was taken (after vigorous shaking). Subsequently, the absorption spectrum was measured in the wavelength range between 200 and 700 nm. An absorption peak was observed at λ = 267 nm for the PY solutions. The solution in contact with the pristine adhesive (control, without PY) was used as a reference. After each measurement, 0.5 mL of distilled water was added into each glass bottle to maintain a constant volume.

The pH measurements were performed at 24 ± 2 °C after the incubation of the specimens with water in the same conditions as for the release experiments for 3 h and 24 h.

### 2.4. Bond Strength Test

Sixty caries-free, freshly extracted, human mandibular molars, were used in this study. The teeth were stored at 4 °C for 24 h. Two sections were made perpendicular to the longitudinal axis of the tooth crown using a saw microtome (Walter EBNER, Le Locle, Switzerland), to obtain dentin discs of 4 mm in thickness. The coronal surface was then hand-polished using a 320-grit silicon carbide paper (Escil, Chassieu, France) for 60 s under continuous water irrigation. The teeth were divided into three groups (20 teeth each) based on the different concentrations of pyrogallol (0%, 0.5%, and 1%) used in this study. The adhesives were applied in the self-etch mode as per the manufacturer’s instructions. A silicone mold (3 mm in diameter) was used to make the resin composite build-ups on the occlusal dentin surface of the specimens using a resin composite Reflectys (ITENA Clinical, Paris, France) [33], which was applied in three layers of 2 mm each. Each layer was light cured for 40 s using an LED curing system (Luxite Lampe LED, ITENA Clinical, Paris, France). Ten specimens of each group were stored in distilled water at 37 °C for 24 h, while the remaining specimens were conserved in water at 37 °C for 6 months.

After each storage period, ten specimens from each group were analyzed. The shear test consisted of applying a shear load to the interface between a cylinder bonded to a dentin disk. The shear load was applied using a metal tap of an Instron dental shear apparatus (Instron standard ISO/TS 11405), which was mounted on an Instron universal tension/compression machine (Instron 3345, High Wycombe, U.K.). The machine was equipped with a 1 kN load cell (Instron 2519-1 kN) and a controller for displacement. The metal tap was initially positioned near the interface without touching the specimen. The force value was then set to zero. The tap displacement was set at a rate of 0.5 mm/min until rupture occurred. Force values were recorded during tests using the Bluehill^®^ universal software. The SBS was measured in megapascals (MPa), which was obtained by dividing the maximum load force (N) at the time of debonding by the bonded area (mm^2^). After the SBS test, an optical numeric microscope (Keyence, Osaka, Japan) was used to investigate the failure mode in each specimen. VHX-5000 software was used to calculate the percentage of each area at 50× magnification to define the type of fracture. The failures were categorized into cohesive, adhesive, and mixed failure modes.

### 2.5. Scanning Electron Microscopy (SEM)

Nine molars were prepared as described in Section 2.4. Three specimens from each group (0%, 0.5%, and 1% PY) were sectioned along the sagittal plane using a diamond-embedded saw mounted on a microtome (Walter EBNER, Le Locle, Switzerland). Subsequently, the resin–dentin interfaces of the specimens were etched using 37% phosphoric acid for 10 s, rinsed for 10 s with distilled water, and immersed in a 2.5% NaOCl solution for 3 min [32]. The specimens were finally rinsed with distilled water and dehydrated in a graded series of ethanol solutions. They were then mounted on aluminum SEM stubs, and sputter-coated with gold–palladium alloys (20/80) using a sputtering device (Hummer JR, Technics, CA, USA). The adhesive layer thickness (five measurements for each section) was determined using a Quanta 250 FEG scanning electron microscope (FEI Company, Eindhoven, The Netherlands) at 10 kV acceleration voltage of the electrons.

### 2.6. Statistical Analysis

The Shapiro–Wilk test was used to verify the normality of the data within all groups. One-way analysis of variance (ANOVA) was assessed using SigmaPlot software (release 11.2, Systat Software, Inc., San Jose, CA, USA) to determine whether significant differences existed in the SBS values (24 h and 6 months), adhesive layer thickness, and antibacterial activity after 24 h. In all tests, a statistical significance level of α = 0.05 was adopted.

## 3. Results

### 3.1. Antibacterial Activity (ADT and DCT)

**Figure 3:**
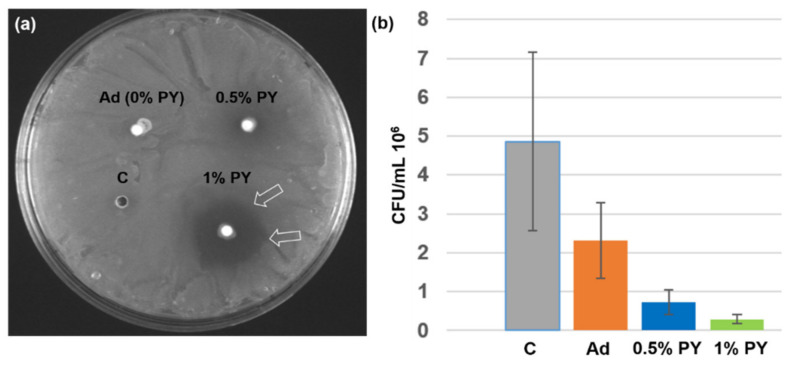
(**a**) Agar diffusion test with the adhesive (Ad), adhesive + 0.5% PY (0.5% PY), and adhesive + 1% PY (1% PY) (white arrows for the inhibition zones). (**b**) Number of CFU of *S. mutans* in the presence of the adhesive (Ad), adhesive + 0.5% PY (0.5% PY), and adhesive + 1% PY (1% PY) after 24 h of incubation at 37 °C. C: bacterial medium.

The agar diffusion tests showed a prominent antibacterial activity of the adhesive doped with 1% PY against *S. mutans*, with inhibition zones of 5 ± 1 mm after 24 h of incubation. Conversely, no inhibition zones were observed for the control and the adhesive doped with 0.5% PY (Figure 3a). The DCT showed that the adhesive doped with 1% PY killed 94% of *S. mutans* when compared with the control group (bacterial medium) (Figure 3b), whilst the adhesive loaded with 0.5% PY destroyed 85% of the *S.mutans* bacteria.

### 3.2. Release Kinetics of the PY and pH Changes

**Figure 4:**
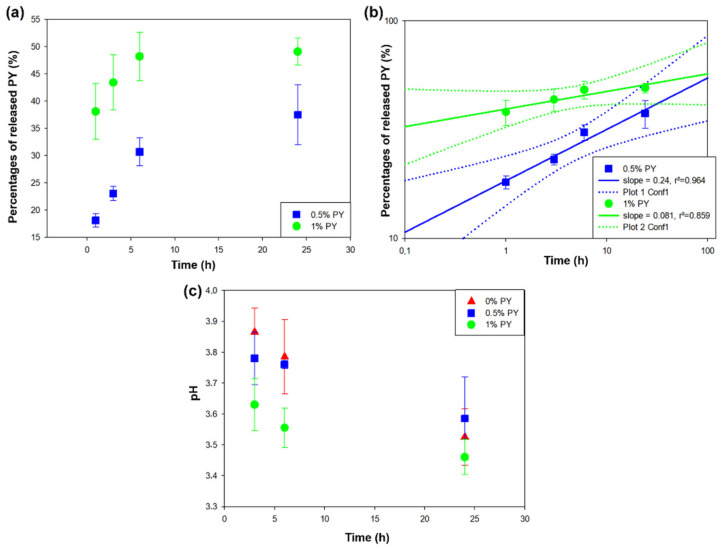
(**a**) Percentages of the PY released from the adhesives doped with 0.5% and 1% PY over time for two independent experiments). (**b**) The data from part (**a**) are plotted in a double logarithmic representation and fitted with straight lines whose slope is given in the inset. The dotted lines correspond to the limits of the 95% confidence intervals. (**c**) pH changes with time of water put in contact with the adhesive (control), adhesive + 0.5% PY, and adhesive + 1% PY. The experimental conditions are indicated in the insets.

After the antibacterial tests, the weight percentages of PY released in water after 1, 3, 6, and 24 h (Figure 4a) were measured; 49% of PY initially present in the adhesive was found in the media of the 1% PY adhesive after 24 h. For the 0.5% PY group, 37% of the PY was released from the adhesive doped with 0.5% PY. The release kinetics was higher for the adhesive doped with 1% PY than for the 0.5% PY. The results show that the higher the concentration of PY that was initially incorporated in the blend, the higher the steady-state concentration of PY released in the water (Figure 4a).

We also aimed to explain the release mechanisms of PY from the adhesive. Unfortunately, our experimental design did not allow for monitoring the release kinetics at small contact times between the material and water because no continuous stirring was performed. The use of models to fit the release kinetics was very sensitive to data acquired over short durations such that it was possible to analyze the data for periods no longer than 1 h. The experimental (Figure 4a) values were plotted on a double-logarihmic scale (Figure 4b) and these fitted reasonably well with the Korsemeyer–Peppas model [34,35]. This model indicated that the fraction of released molecules scaled with time as t*^α^*, where the value of *α* can give some insight into the release mechanism. In our case, the slope of the straight line in a double-logarithmic plot directly gave the value of *α*. However, in the case of the adhesive incorporated with 0.5% PY, *α* was 0.25, whilst the value of *α* was 0.08 in the case of the adhesive blended with 1% PY. This means that the release process was certainly governed by a single diffusion process for which *α* < 0.5 was expected [35]. Note also that even though the release rate in the case of adhesive blended with 1% PY was higher at *t* < 1 h than for the adhesives containing 0.5 % PY, at *t* > 1 h, the last one had a faster release than the adhesive doped with 1% PY. This factor might be due to a release of PY in a slightly aggregated form in the case where it was embedded at a 1% mass fraction. Those small aggregates may originate from the fact that the liquid constituting the prepolymerized adhesive was a less suitable solvent than water for the solubilization of PY. The hypothetical release in the form of small aggregates will be validated in future investigations.

In addition, the pH of water in contact with the adhesive +1% PY was slightly lower than the other groups (Figure 4c) after 3, 6, and 24 h.

### 3.3. Shear Bond Strength (SBS)

**Figure 5:**
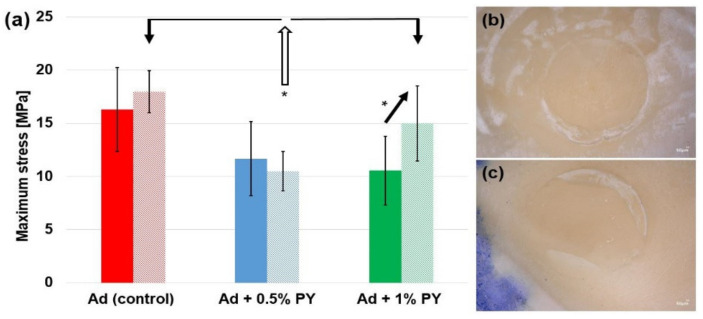
(**a**) Evolution of the bond strength for the universal adhesive (Ad “control”) and the universal adhesive modified with 0.5% (Ad + 0.5% PY) and 1% PY (Ad + 1% PY) at 24 h (dark-colored bars) and 6 months (slightly colored bars). (* *p* < 0.05). (**b**,**c**) Representative images obtained with an optical microscope (×50 magnification): (**b**) adhesive failure and (**c**) mixed failure.

The bond strength values of the control adhesive and those of the adhesives doped with 0.5% and/or 1% PY are shown in Figure 5a. No significant difference was found between the three groups after 24 h of storage period (*p* = 0.054). However, after 6 months of storage in water, the bond strength of the adhesive doped with 0.5% PY was significantly lower than the bond strength of the control group and the adhesive doped with 1% PY (*p* < 0.05). The specimens created with the adhesive doped with 1% PY demonstrated a significantly higher bond strength after 6 months of storage than the same group at 24 h (*p* < 0.05). A prevalent mixed failure (Figure 5c) was observed for the unmodified adhesive group (24 h and 6 months) and the adhesive doped with 1% PY after 6 months. A predominant adhesive failure was observed for both modified groups with PY at 24 h and the 0.5% PY at 6 months.

### 3.4. SEM Observations

**Figure 6:**
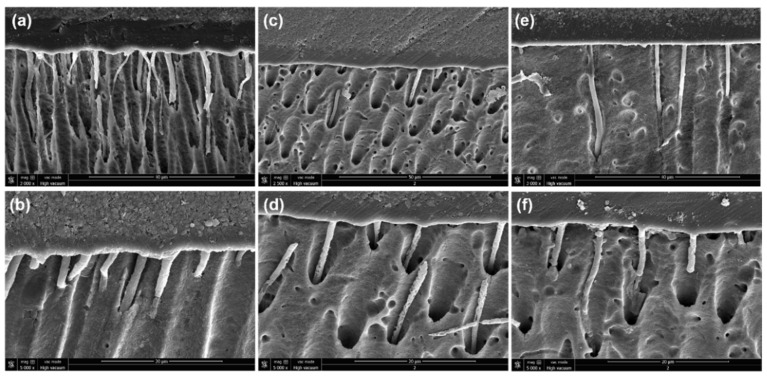
Representative scanning electron microscopy micrographs (×2000 and ×5000 magnifications) demonstrating the adhesive layer thickness: (**a**,**b**) adhesive group (control), (**c**,**d**) adhesive + 0.5% PY, and (**e**,**f**) adhesive + 1% PY.

There was no clear morphological difference between the interfaces created with the tested adhesive. Moreover, the mean thickness of the control adhesive layer (6.5 ± 0.67 µm) and the adhesive layer doped with 0.5% (8.1 ± 3.2 µm) and/or 1% (7.3 ± 1.8 µm) of PY was between 4 and 11 µm, as measured using SEM (Figure 6). No significant difference was found between the layer thicknesses for the different tested groups (*p* > 0.05). The infiltration depth of the unmodified adhesive and the adhesives doped with PY were observed. The infiltration depth of the inter-diffusion layer was between 5 and 33 μm into the dentinal tubules for all tested adhesives. The addition of PY to adhesive materials had no impact on the adhesive layer thickness or the tag length.

## 4. Discussion

The incorporation of PY into universal adhesives may influence their bonding performance to dentin and ameliorate the antibacterial effects against cariogenic species, such as *S. mutans*. An ideal restorative material should possess some antibacterial properties, along with good mechanical properties and bonding stability over time [1,21,36]. Unfortunately, the resin adhesive systems that are currently available on the market do not completely fulfill all these requirements.

The DCT and ADT tests performed in this study demonstrated that the presence of 1% PY within the polymer matrix of resin adhesive systems may confer specific antibacterial effects against *S. mutans*. However, when using a PY concentration of 0.5%, only a lower antibacterial effect in DCT was observed compared to the experimental resin adhesive containing 1% of PY (Figure 3b). Conversely, no antibacterial activity was observed when the 0.5% PY was tested for ADT (Figure 3a). This outcome could be due to the lower PY liberation from the adhesive doped with 0.5% PY (Figure 4a). Therefore, the first null hypothesis must be rejected.

A brown color was observed around the 1% PY in ADT due to oxidation of the polyphenols in the growth media and the higher release of PY from the resin. However, the adhesive devoid of any PY (control group) had some sort of antibacterial activity against *S. mutans* when compared to the bacterial medium. This antibacterial activity could be related to the acidity of the 10-MDP functional monomer or to the elution of some unpolymerized components present in the adhesive system, which are usually toxic to bacteria [37]. In the literature, it is possible to see that other polyphenols, such as epigallocatechin-3-gallate (EGCG) and catechin, were added to dental adhesives to promotes better antibacterial activity [38,39] and bonding durability [40].

The release of PY induced a slight decrease in the pH and this was more pronounced for the 1% PY polymer blend than the one containing 0.5% PY (Figure 4c). The higher release from the specimens of adhesive doped with 1% PY could explain such a lower pH of water when compared to the pH of the adhesive system containing 0.5% PY. However, in the case of adhesive blended with 1% PY, about 50% of the PY that was initially incorporated in the blend was released into the water after 6 h and the same percentage of PY was found in the water after 24 h. This suggests that there were two populations of incorporated PY in the adhesive structure. Therefore, the incorporated PY in the adhesive–polymer structure had a dual role: about 50% was incorporated into the adhesive structure and 50% was free to enhance the antibacterial effects.

However, the bond strength and stability of resin adhesives bonded to dental tissues are important aspects to attain optimal dental restorations in an oral cavity [1,3,5,21]. Indeed, several studies [41,42,43,44,45] reported a severe degradation of the polymer–dentin interface over time due to a synergic hydrolytic degradation of the polymer matrix within the hybrid layer and to the enzymatic degradation of poorly infiltrated demineralized dentin collagen fibrils through activated host metalloproteinases (MMPs) and cysteine-cathepsins (CTs). In this study, the SBS test was used to evaluate the bond strength of the control and the adhesives doped with PY to avoid artifacts during sectioning procedures that may occur for the preparation of microtensile beams (stick of 1 mm^2^) [33]. Sano et al. [46] reported that the microtensile test may be better than the SBS test to analyze the bond strength of an adhesive, whilst several studies reported that the significant factor for bond strength is the adhesive system used regardless of the testing model used [47].

This study demonstrated that the control adhesive without PY preserved the bond strength to dentin after 6 months. These latter results are in agreement with similar findings reported in a previous study [33]. Such outcomes could be in part related to the self-etching application mode and to the 10-MDP present within its formulation [33]. Conversely, despite the lower bond strength values, the addition of PY (0.5% and 1%) showed no significant difference between the adhesive after 24 h. These results could be associated with a low degree of conversion after 24 h [48]. The adhesive doped with 1% PY increased the bond strength after the storage period, while the 0.5% PY preserved the bond strength, but with significantly lower values compared to the control adhesive with no PY. Therefore, the second null hypothesis must be rejected. The same observations were reported by previous studies when quercetin was used in resin adhesive systems [40,48]. Moreover, such an outcome may be a consequence of the reaction of some antioxidants that promoted a prolonged late polymerization and thus increasing the resistance of the bonding interface to degradation. Indeed, such a late polymerization may have also influenced the bond strength after the 6-month storage period [48]. It is well known that the bond strength of an adhesive resin can be reduced by the presence of oxygen via the inhibition of the polymerization of resin monomers [49]. Antioxidants decrease the rate of free oxygen radicals; this favors the polymerization process of the bonding materials and enhances the bonding performance of adhesive systems [49]. The results of adhesives doped with PY obtained in the present study could be related to the antioxidant properties of pyrogallol. Yang et al. [50] reported a better sealing ability of adhesive resin modified with quercetin to dentin. These findings could be attributed to the protective effect of quercetin on dentin collagen. Therefore, some antioxidants could crosslink collagen proteins via the formation of hydrogen bonds and increase the resistance of dentin to degradation.

SEM observations were made in order to verify any changes at the polymer–dentin interface, especially with the adhesive doped with PY. The thickness of the adhesive layer of the tested adhesive doped with different weight percentages of PY presented no remarkable changes under SEM analysis compared to the unmodified adhesive (control group). All the adhesives produced a hybrid layer, some resin tags that infiltrated into dentinal tubules, and a continuous adhesive layer without voids. In accordance, Fonseca et al. [29] reported a thin adhesive layer and tags infiltration into dentinal tubules at 24 h after the use of an adhesive modified with different weight percentages of ECGC. All adhesive groups dissolved the smear layer and allowed adhesive tags to infiltrate into the dentin structure. Therefore, the addition of PY to adhesive materials did not alter the creation of resin tags, which infiltrated dentinal tubules for several microns. The infiltration depth into dentinal tubules of the unmodified adhesive and the adhesive doped with both PY percentages appeared to be similar under SEM observations.

The chemical interaction between the PY and adhesive deserves further investigation. Therefore, future studies should be performed to evaluate the solubility, degree of conversion, shelf-life stability, and polymerization rate of the adhesive modified with PY. Moreover, additional studies on cytotoxicity are recommended in order to analyze the biocompatibility of these PY-loaded adhesive systems.

## 5. Conclusions

Within the limitations of this study, we concluded that the incorporation of 1% PY into universal adhesive systems could enhance the antibacterial activity and preserve the bond strength to dentin over time.

## Figures and Tables

**Table 1:** Universal adhesive, chemical composition, application process, and manufacturing.

Material	Composition	Applications
Iperbond Max (Itena Clinical, Paris, France)	10-MDP, 4-META, methacrylates, photo-initiators, ethanol, water, fumed silica	Apply (20 s) Wait until the solvent had completely evaporated (20 s) Dry (5 s) Light cure (10 s)

## Data Availability

Not applicable.
